# Role of gut microbiota and its metabolites in diabetic cardiomyopathy: from pathogenesis to interventions

**DOI:** 10.3389/fcvm.2025.1677684

**Published:** 2025-09-22

**Authors:** Zheng Ji, Xinrou Yu, Haodi Gu, Ping Wang, Liping Meng, Hui Lin, Haitao Lv

**Affiliations:** ^1^ Department of Cardiology, Shaoxing People’s Hospital, Shaoxing, Zhejiang, China; ^2^ School of Medicine, Shaoxing University, Shaoxing, Zhejiang, China; ^3^ Department of Cardiology, Zhuji Affiliated Hospital of Wenzhou Medical University, Shaoxing, China; ^4^ Department of Cardiology, The Affiliated Lihuili Hospital of Ningbo University, Ningbo, Zhejiang, China

**Keywords:** diabetic cardiomyopathy, gut microbiota, metabolites, short-chain fatty acids, gut-heart axis

## Abstract

Diabetic cardiomyopathy (DCM), a prevalent cardiovascular complication and the principal driver of mortality among patients with diabetes, represents a significant clinical challenge. The gut microbiota, which reside a complex ecosystem within the human intestinal tract, play a fundamental role in host metabolism and systemic physiology. Mounting evidence underscores a critical link between gut microbial dysbiosis, microbial-derived metabolites, and DCM pathogenesis mediated through the gut-heart axis. This comprehensive review systematically synthesizes the current research elucidating the multifaceted interplay between the gut microbiota, their bioactive metabolites (e.g., short-chain fatty acids, bile acids, and branched-chain amino acids), and the development and progression of DCM. By critically evaluating the mechanisms underlying the gut-heart crosstalk, we provide novel insights into the etiopathogenesis of DCM. Furthermore, we evaluated emerging therapeutic strategies aimed at mitigating DCM by targeted modulation of the gut microbiota and their metabolic output, highlighting promising avenues for future research and clinical translation.

## 1 Introduction

Diabetic cardiomyopathy (DCM) is a distinct clinical manifestation of cardiovascular disease and is the principal cause of mortality in individuals with diabetes (1). In its initial stages, metabolic disturbances precipitate alterations in the cardiac structure and function, characterized by impaired insulin signaling, insulin overexpression, disrupted cellular glucose utilization, increased myocardial uptake of non-essential fatty acids, and mitochondrial dysfunction (2). These physiological and metabolic anomalies contribute to cardiac remodeling, myocardial fibrosis, diastolic dysfunction, and ultimately to a reduction in ejection fraction in patients with diabetes (3, 4). DCM is a significant cause of mortality in patients with diabetes, independent of preexisting factors that compromise the cardiac ejection fraction. The incidence of heart failure is at least 2–5 times greater in individuals with diabetes than in those without diabetes (5, 6). Currently, there are no targeted pharmacological treatments for DCM, highlighting the need to identify novel therapeutic strategies for DCM management.

The gut microbiota constitutes a community of microorganisms residing in the human gastrointestinal tract that plays a crucial role in the metabolism of various nutrients. It has been described as an acquired “new organ” and the “second genome,” both of which are integral to maintaining health (7). A significant difference in the relative abundance of microbiota exists between individuals with diabetes and healthy controls (8). Consequently, the metabolic potential of the gut microbiota is considered a contributing factor in the development of DCM through the gut-heart axis (9). The microbiota engages with the host via multiple pathways, such as the short-chain fatty acids (SCFAs) pathway, branched-chain amino acids (BCAAs) pathway, and the primary and secondary bile acids pathways (9). This study systematically reviewed the role of the gut microbiota and its metabolites in DCM, providing a theoretical foundation for targeting the gut microbiota as a novel therapeutic approach for DCM.

## 2 Pathologic mechanisms of DCM

DCM is a heart disease associated with diabetes, characterized by metabolic disturbances, fibrosis, and impaired systolic and diastolic functions in cardiomyocytes. The prevailing hypothesis suggests that metabolic abnormalities induced by hyperglycemia and insulin resistance result in increased oxidative stress, inflammatory responses, and fibrosis within cardiomyocytes. Insulin resistance is considered a pivotal mechanism in the pathogenesis of DCM. Given that the myocardium is one of the most metabolically active tissues in the body, it utilizes a diverse array of substrates for energy production, primarily comprising approximately 70% free fatty acids (FFA) and 30% glucose (10).

**Figure 1:**
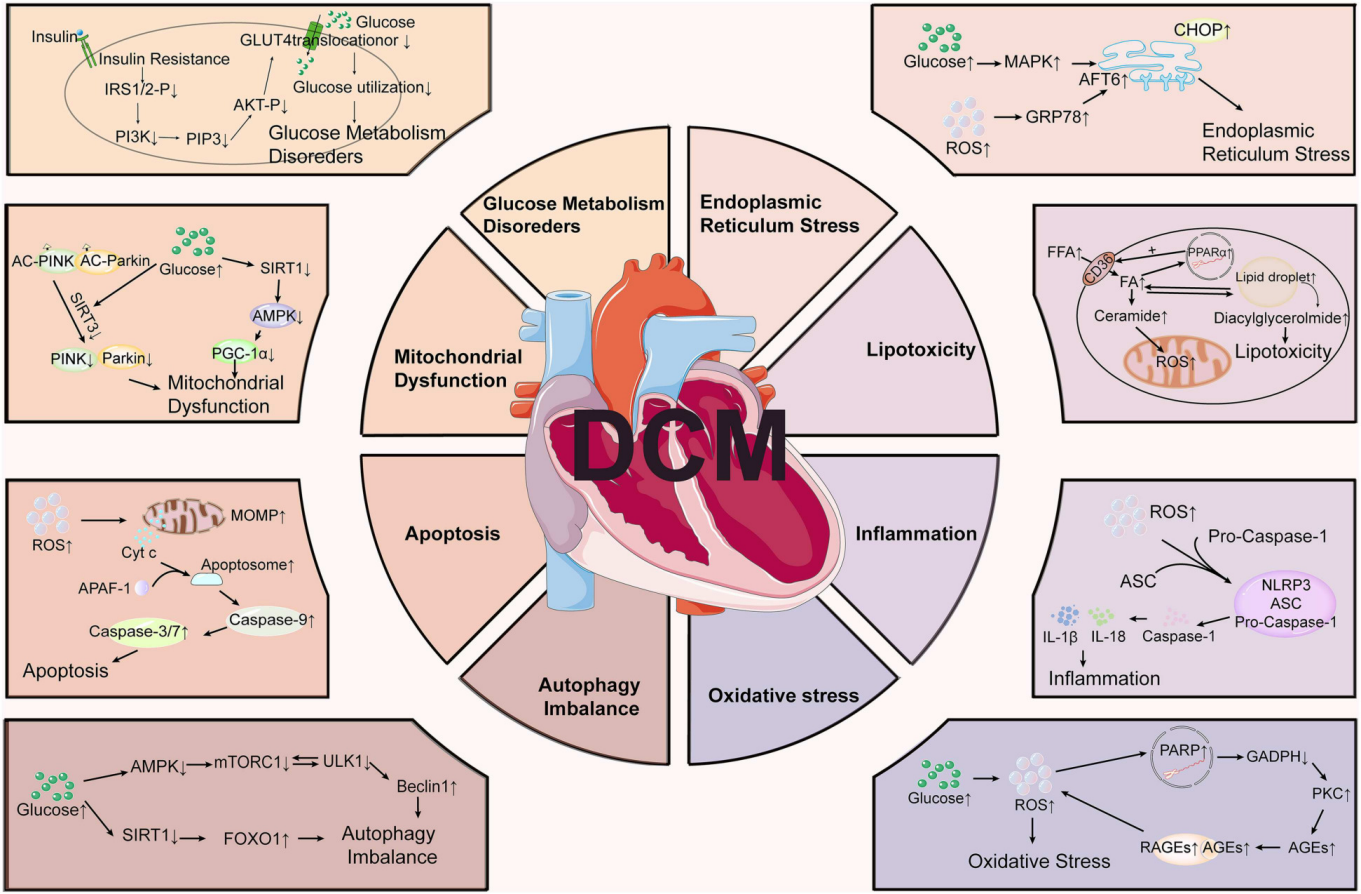
Molecular mechanisms involved in the pathogenesis of diabetic cardiomyopathy (DCM). DCM involves multiple pathological processes, including glucose metabolism disorders, mitochondrial dysfunction, endoplasmic reticulum stress, lipotoxicity, inflammation, oxidative stress, apoptosis, and autophagy imbalance.

Extensive research has shown that many molecular proteins and signaling pathways play important roles in the development of DCM (Figure 1). In the context of diabetes, there is a notable reduction in glucose transporter proteins, specifically Glut-1 and Glut-4 (11), which impairs the myocardium's capacity to efficiently utilize glucose. This reduction diminishes a vital energy source, compelling the myocardium to rely increasingly on fatty acid metabolism. Concurrently, insulin resistance and enhanced lipolysis elevate FFA levels in the bloodstream, which can induce significant cytotoxic effects. Furthermore, the augmented β-oxidation of FFAs within cardiomyocytes contributes to electron leakage in the electron transport chain (ETC), facilitating the accumulation of reactive oxygen species (ROS). This accumulation results in structural and functional abnormalities in cells (12–15). ROS also activate a variety of signaling pathways, such as NF-κB, p38 MAPK, and JNK (16, 17). The activation of these pathways can further amplify inflammatory responses and apoptosis, thereby exacerbating lipotoxicity, playing a critical role in the cardiac dysfunction associated with DCM. Additionally, insulin resistance maintains the myocardium in a state of persistent hyperglycemia with excess circulating glucose-activating polyol protein kinase (PKC) through the hexosamine pathway. PKC activation facilitates the formation of advanced glycosylation end-products (AGEs) (18). The interaction between AGEs and the receptor for AGEs (RAGE) results in elevated oxidative stress, which enhances the production of ROS and activates inflammatory mediators such as intercellular adhesion molecule 1 (ICAM-1), vascular cell adhesion molecule 1 (VCAM-1), tumor necrosis factor-alpha (TNF-α), and interleukin-6 (IL-6), consequently leading to cellular damage (19). Additionally, PKC activation can further stimulate NF-κB signaling pathway, thereby promoting the expression of inflammatory mediators and exacerbating the inflammatory response and cellular injury (20). Consequently, insulin resistance induces sustained oxidative stress in cardiomyocytes, a condition that may inflict damage on the mitochondria and DNA, initiate lipid peroxidation in cell membranes, and activate multiple cell death pathways.

The effect of DCM on cardiac health extends beyond cardiomyocytes and encompasses various heart structures and functions. Notably, diabetes-induced small-vessel vasculopathy compromises the blood supply to the cardiac microvasculature, precipitating myocardial ischemia and dysfunction (21). Impaired endothelial cell function is a critical factor in microvascular dysfunction. This impairment arises from oxidative stress and the accumulation of AGEs due to chronic hyperglycemia, which directly damages endothelial cells and affects the diastolic function of microvessels (22). Consequently, compromised endothelial cell function results in decreased nitric oxide release and increased endothelin-1 release, leading to abnormal microvascular contractions. The resultant impaired vasodilation adversely affects cardiac blood supply, ultimately diminishing cardiac function (23).

Furthermore, sustained hyperglycemia stimulation can also result in the overactivation of cardiac fibroblasts (CFs) and induce their differentiation into myofibroblasts, resulting in myocardial extracellular matrix (ECM) imbalance and myocardial fibrosis (24). Activation of TGF-β/Smads and AMPKα signaling pathway is able to promote the proliferation, activation and collagen production of CFs to induced cardiac fibrosis in DCM (25). Cardiac hypertrophy and altered collagen subtype deposition are key features of maladaptive remodeling in DCM. Accumulating evidence indicates that microbial metabolites such as trimethylamine N-oxide (TMAO) promote hypertrophy and fibrosis via TGF-β/Smad and MAPK pathways (26), whereas SCFAs and bile acids may exert protective, antihypertrophic effects through epigenetic and GPCR-mediated signaling (27–29). These findings support a causal link between gut microbiota dysbiosis and hypertrophic remodeling, further reinforcing the gut–heart axis in DCM pathogenesis.

In summary, the molecular mechanisms underlying DCM are highly complex and involve multiple cardiac cell types. These include cardiomyocyte injury, metabolic abnormalities, endothelial dysfunction, aberrant fibroblast activation, and functional alterations in other cell types. An in-depth study of the pathological mechanism and related molecular pathways of DCM, as well as a summary of the relevant achievements in the treatment of current research are expected to offer valuable insights for the prevention and therapy of DCM.

## 3 The role of gut bacterial and its metabolites in the development of DCM

### 3.1 Composition of gut microbiota

The human gut microbiota is a complex and diverse community that plays a crucial role in various physiological processes, including nutrient absorption, immune regulation, and maintenance of defense barriers (30). This vast microbial ecosystem comprises hundreds of millions of microorganisms, including bacteria, fungi, and viruses (31). The human gastrointestinal tract harbors approximately 1,000–1,150 bacterial species distributed across seven phyla (32). More than 99% of these microorganisms belong to Firmicutes, Actinobacteria, Bacteroidetes, and Ascomycetes, whereas Clostridia, Archaea, Deferobacteria, and Spirochaetes constitute less than 1% (30). Extensive research has demonstrated a strong association between the gut microbiota and the preservation of host health and pathogenesis of diseases (33). Notably, an increased ratio of Firmicutes to Bacteroidetes is indicative of a disruption in gut microbial homeostasis, which is potentially linked to certain pathological conditions, whereas a decreased F/B ratio is associated with reduced levels of beneficial gut flora and an increase in potentially pathogenic bacteria (34). This ratio is subject to alterations caused by factors, such as dietary changes, physical activity, antibiotic use, and other external influences.

### 3.2 Dysbiosis of gut microbiota and DCM

**Figure 2:**
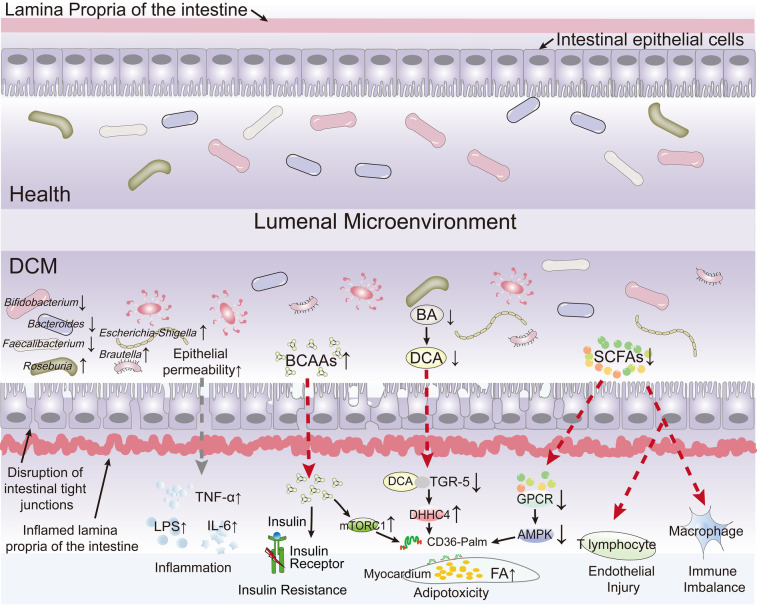
The current research elucidating the multifaceted interplay between the gut microbiota, their bioactive metabolites, intestinal barrier function, and the development and progression of diabetic cardiomyopathy (DCM). The diagram highlights how the gut microbiota and their bioactive metabolites interactions influence the development and progression of DCM by depicting pathways leading to increased epithelial permeability, inflammation, insulin resistance, adipotoxicity, endothelial injury, and immune imbalance. Key microbial species (e.g., Bifidobacterium, Bacteroides, Escherichia/Shigella, Faecalibacterium, Roseburia) and their roles in health vs. disease states are shown, along with molecular mediators like LPS, TNF-α, IL-6, TGR-5, and AMPK.

An expanding body of evidence indicates that modifications in gut microbial species, their abundance, and shifts in colonization patterns are implicated in the pathogenesis of obesity, diabetes, cardiovascular diseases, and metabolic and neurodegenerative disorders (35–37). Disruption in the proportion of gut microbiota can compromise the integrity of the intestinal epithelium and increase its permeability, resulting in metabolic endotoxemia (38, 39). This study systematically reviews current research elucidating the multifaceted interplay between the gut microbiota, their bioactive metabolites (e.g., short-chain fatty acids, bile acids, and branched-chain amino acids), intestinal barrier function, and the development and progression of DCM (Figure 2).

Commonly reported findings suggest that Bifidobacterium spp., Anaplasma spp., E. Faecalibacterium spp., Ackermann spp., and Rothbaryomyces spp. are negatively associated with type 2 diabetes (40–43), whereas Ruminalococcus spp., Clostridium spp., and Brautella spp. are positively associated with the condition (44). According to a study by Tsai et al. (45), an analysis of gut microbiota species abundance and echocardiographic data from 155 patients with type 2 diabetes revealed associations between the phylum Firmicutes, the genera Mycobacterium anisopliae, the F/B ratio, and Mycobacterium spp. with structural cardiac changes as well as systolic and diastolic dysfunction in these patients. SCFAs-producing bacteria, including Bifidobacterium (45–47), Roseburia (48, 49) and Faecalibaculum prausnitzii (42, 50) was consistencily reported significantly reduced in patients with in type 2 diabetes patients and in patients with heart failure, as well as in diabetic animal models. On the contrary, Escherichia-Shigella, which was identified as a harmful bacterium, was increased in patients with type 2 diabetes and associated with the risk of cardiovascular complications (51, 52). Zhao et al. found in failing hearts that dysbiosis led to the exhaustion of butyric acid-producing bacteria, inhibited fatty acid oxidation in myocardial cells, and turned to rely on low-efficiency glycolysis, forming an “energy hunger” state, which further disrupted myocardial metabolism (53). Additionally, mycobacteriophages, thick-walled mycobacteriophages, and Mycobacterium spp. were positively correlated with left ventricular ejection fraction. Furthermore, low levels of the Firmicutes phylum have been significantly associated with an increased risk of left ventricular hypertrophy (45). Kummen et al. (47) also reported a reduced abundance of Bifidobacterium spp. in patients with heart failure compared to healthy individuals. Although significant gut microbiota dysbiosis is observed in patients with DCM, the specific gut-heart axis pathways through which dysbiosis disrupts myocardial metabolism and compromises cardiac function, fueling DCM advancement, require deeper mechanistic investigation. Table 1 systematically delineates the human-versus-murine alterations in gut microbiota associated with DCM and provides a comparative analysis of inter-species concordance and discordance. 

**Table 1:** The characteristically changed gut microbes in human or animal models with diabetic cardiomyopathy (DCM).

Bacterial genus	The changing trend in DCM	Human Research	Animal Model Research	Analysis of consistency and difference
Bacteroides	Increase or decrease	It was decreased in patients with chronic heart failure by Zhang et al (40) and Mirhosseini et al (41), but increased in the study reported by Huang et al (36); In patients with T2DM, Bacteroides are deceased and negatively correlated with T2DM (37).	The abundance of Bacteroides was deceased in the STZ-induced diabetic mice or rat model (16, 90).	Notable discrepancies are observed, with inconsistent directions of change. These variations may be associated with different stages in the onset and progression of DCM, warranting further investigation
Bifidobacterium	Decrease	Low abundances of Bifidobacterium was observed in T2DM patients (45) and in patients with heart failure (46).	Bifidobacterium was decrease in DCM mice and abundant by myricetin treatment (47).	High consistency. As another important genus of SCFAs-producing bacteria, its reduction has been observed in both human and animal models.
Lactobacillus	Decrease	The abundance is relatively reduced in the cohort of patients with chronic heart failure (99).	The abundance of Lactobacillus was significantly reduced in STZ-induced diabetic mice (69).	The consistency is relatively high. The reduction of Lactobacillus is a core feature of the dysbiosis related to diabetes.
Roseburia	Decrease	The abundance is decreased in patients with T2DM (51).	The abundance is decreased in the rat model of T2DM (49).	High consistency. As another important genus of SCFAs-producing bacteria, its reduction has been observed in both human and animal models.
Faecalibacterium prausnitzii	Decrease	Its abundance is significantly reduced in patients with T2DM complicated with cardiac insufficiency (45).	It was reduced in prediabetic and diabetic mice. Faecalibacterium and its extracts lower blood glucose and HbA1c levels, and improved glucose response in mice (50).	High consistency. Its abundance has been observed to decline in both human and animal models. Its reduction is an important feature of DCM.
Escherichia-Shigella	Increase	It is associated with the risk of cardiovascular complications in patients with diabetes (51, 52)	In the DCM model, the amplification of LPS-rich strains promotes systemic inflammation (29).	High consistency. The increase of this genus is a key indicator of the pro-inflammatory state and is closely related to the inflammatory pathology of DCM in both human and animal models

T2DM, type 2 diabetes mellitus; LPS, lipopolysaccharide; STZ, streptozotocin; SCFAs, short-chain fatty acids.

### 3.3 The role of gut microbial metabolites in the development of DCM

#### 3.3.1 Bile acids

Bile acid metabolism is integral to the pathophysiology of cardiovascular and metabolic disorders in patients with diabetes who experience myocardial damage. In db/db mice (a leptin receptor-deficient DCM model), a decrease in Firmicutes and an increase in Helicobacter were observed. Treatment with Scutellariae Radix and Paeoniae Radix Alba restored the gut microbiota equilibrium by modulating myocardial glycerophospholipid and arachidonic acid metabolism via bile acid/taurine pathways. This attenuates cardiac dysfunction, hypertrophy, and fibrosis in DCM (54).

Changes in plasma bile acid profiles in patients and animal models with diabetes suggest an association between the bile acid-G protein-coupled receptor 5 (TGR5) pathway and diabetes (55). TGR5 is a member of the protein-coupled receptor family and serves as a bile acid membrane receptor with a significant regulatory function in glucose metabolism (56). TGR5 is extensively expressed in various human tissues, including the gallbladder and intestines, and its mechanism of action involves multiple signaling pathways (57). Activation of TGR5 influences energy metabolism and regulates intestinal motility. Additionally, bile acid metabolism, a crucial component of bile, is modulated by gut microbiota. The gut microbiota metabolizes bile acids secreted into the duodenum into secondary bile acids (58), and alterations in the composition of these secondary bile acids reciprocally affect the distribution of the gut microbiota (59). According to Wang H et al. (60), in patients with diabetes, myocardial damage is associated with decreased levels of bile acids, particularly those that preferentially bind to TGR5 receptors, such as deoxycholic acid, which are positively correlated with cardiac function. The absence of TGR5 augments the uptake of fatty acids by the heart, leading to lipid accumulation within cardiac tissue. This phenomenon was attributed to the loss of TGR5, which resulted in increased DHHC4-mediated palmitoylation of CD36. This modification promotes the localization of CD36 to the plasma membrane, thereby enhancing the myocardial uptake of fatty acids (60). The TGR5-DHHC4 pathway is instrumental in regulating cardiac fatty acid uptake, underscoring the deleterious effects of TGR5 deficiency in patients with DCM. Consequently, bile acid metabolism plays a critical intermediary role in linking TGR5 to the gut microbiota (55).

#### 3.3.2 Short-chain fatty acids

Short-chain fatty acids (SCFAs), including butyrate, acetate, and propionate, are predominantly generated during bacterial fermentation of indigestible carbohydrates in the colon (61). SCFAs play a crucial role in modulating the intestinal barrier integrity, thereby exerting anti-inflammatory effects (62). They influence immune cell function and reduce the levels of pro-inflammatory mediators, thus contributing to a balanced immune response (62). Myricetin can improve cardiac function in mice with DCM by restoring gut microbiota homeostasis, especially for SCFA-producing bacteria, including Roseburia, Faecalibaculum, and Bifidobacterium (47). Specifically, butyrate regulates the intestinal macrophage function by inhibiting histone deacetylases (HDACs), which are essential for maintaining intestinal immune homeostasis and mitigating chronic inflammation, thereby supporting intestinal health and function (63). Recent studies have indicated that propionate enhances vascular endothelial function by reducing the number of inflammatory cells such as T helper 17 cells and effector memory T cells. This reduction aids in maintaining a normal vascular endothelial status and blood flow, consequently diminishing cardiovascular damage (64, 65). Acetate, on the other hand, protects the heart by modulating genes associated with cardiac fibrosis and cardiac hypertrophy, and a high-fiber diet and acetic acid supplementation reduced the expression of nuclear factor κB (Nf-kB), nitrogen oxide reductase 2 (Nox2), and fibroblast growth factor 21 (Fgf21), decreased inflammation, and reduced oxidative stress, which resulted in significantly significant reductions in systolic and diastolic blood pressure, cardiac fibrosis and left ventricular hypertrophy (66, 67). SCFAs also slowed the development of DCM by binding to G protein-coupled receptors, enhancing GLP-1 release, and improving insulin sensitivity (68). Although gut microbiota-derived SCFAs have shown great potential in alleviating DCM, direct evidence that regulation of the gut microbiota influences specific SCFAs levels to improve cardiac function is still lacking. Future research should focus on the protective effects of short-chain fatty acid-producing probiotics, such as Lactobacillus spp. (69) and Bifidobacterium (70), against DCM, along with their therapeutic potential as probiotics to mitigate diabetes-related complications.

#### 3.3.3 Branched-chain amino acid metabolism

Branched-chain amino acids (BCAAs) are comprised of three essential amino acids: leucine, isoleucine, and valine. Following ingestion, proteins are metabolized to BCAAs in the intestine (71–73). Several studies have indicated that elevated levels of BCAAs are present in the blood of obese and insulin-resistant humans and rodents (74–76). Notably, significantly increased levels of cardiac BCAAs have been observed in the left ventricular samples of patients with DCM. This accumulation of BCAAs has been associated with impaired insulin signaling pathways, as evidenced by an increase in the upstream regulator, phosphorylated p70S6 kinase (P-p70S6K), and a decrease in the downstream regulatory factors AKT and phosphorylated glycogen synthase kinase 3 beta (P-GSK3β) (77, 78). The presence of acetolactate synthase-positive S. aureus correlates with BCAA levels and is associated with increased fasting blood glucose (FBG) and insulin resistance in patients with type 2 diabetes (79). When BCAA catabolism is reduced, intracellular BCAA levels increase, especially leucine and isoleucine from bacterial sources accumulate in the myocardium (80), perpetuating the activation of the mTORC1-SREBP1 signaling pathway. This activation promotes lipogenesis while inhibiting lipolysis, leading to cardiac lipotoxicity and the exacerbation of myocardial fibrosis (81). Pyridostigmine was found to restore gut microbiota homeostasis, decrease the abundance of BCAA-producing microbes, and improve the intestinal barrier to reduce circulating BCAA levels, thus exerting protective effects against DCM (82). In addition, restricting dietary BCAAs restored glucose tolerance and insulin sensitivity in HFD-fed mice (83).

However, the oral administration of BCAAs (such as isovalerate, 2-methylbutyrate, and isobutyrate) inhibits lipid accumulation and macrophage foam cell formation, thus attenuating atherosclerosis (84) and aging (85). In high-fat diet-induced obese mice, the long-term effects of BCAAs restriction ameliorated HFD-induced gut microbiota disorder by reducing the abundance of obesity-linked bacteria, such as Lactococcus and Oscillibacter (86). This contradictory phenomenon may be attributed to the intervention time and dosage of the BCAAs used for the aforementioned metabolic diseases. One hypothesis is that dietary BCAAs may exert beneficial effects by supporting myocardial energy metabolism under stress, whereas bacterial-derived BCAAs may contribute to metabolic overload and lipotoxicity through chronic activation of the mTORC1 pathway (87). Moreover, circulating BCAA levels may differentially impact cardiac physiology depending on the host's metabolic state: in insulin-resistant individuals, elevated BCAAs exacerbate metabolic inflexibility and mitochondrial dysfunction, while in nutrient-deprived or failing hearts, moderate supplementation may provide essential substrates for protein synthesis and energy support (88). Supporting this context-specific view, human dietary intervention studies have shown that BCAA restriction can improve insulin sensitivity and metabolic health. For example, Cummings et al. demonstrated that dietary BCAA restriction improved glucose homeostasis and reduced body weight in obese adults, suggesting that excessive BCAA intake may be detrimental in the setting of metabolic syndrome (83). Conversely, experimental models of heart failure suggest that insufficient BCAA supply may impair cardiac protein turnover and worsen contractile dysfunction. Taken together, these findings indicate that the impact of BCAAs on DCM is likely dose-dependent, source-specific, and modulated by host metabolic state, underscoring the importance of personalized dietary and microbiota-targeted strategies.

In summary, current evidence indicates that BCAAs are closely related to the progression of DCM and that reducing the intake of BCAAs may help improve DCM.

### 3.4 Intestinal barrier function and DCM

The intestinal barrier is composed of the intestinal epithelium, mucosal layer, and underlying immune system (89). Intestinal epithelial cells establish a physical barrier that delineates the intestinal lumen from the underlying tissues, thereby preventing the translocation of harmful substances, such as endotoxins and pathogens, from the gut into the circulatory system (90). This barrier also plays a crucial role in modulating the immune response (91). The integrity of the intestinal barrier is maintained by tight junction proteins such as occludins and claudins, which regulate the permeability of the epithelial layer. In mice with DCM, the ability of the gut microbiota to support barrier function is impaired, leading to increased intestinal permeability (92). Disruption of these tight junctions results in increased intestinal permeability, commonly referred to as “leaky gut,” allowing harmful substances to enter the bloodstream and subsequently trigger a systemic inflammatory response (93). In individuals with diabetes, persistently elevated blood glucose levels and metabolic abnormalities frequently lead to alterations in the intestinal barrier function, which in turn leads to chronic inflammation. This inflammation adversely affects cardiac tissue, accelerating myocardial damage and cardiac decline, thereby exacerbating DCM symptoms (94). Furthermore, it was demonstrated that the enhancement of intestinal barrier function can be beneficial for DCM through reduction of endotoxins, such as lipopolysaccharide (LPS), and inflammatory mediators (TNF-α and IL-6 etc.). These mechanisms contribute to the attenuation of systemic inflammation and the deceleration of intestinal permeability, thereby potentially ameliorating DCM (47).

## 4 Adjunctive therapies based on gut microbiota

Modulation of the gut microbiota, inspired by the gut-heart axis (95), may effectively improve cardiac function in patients with DCM. This suggests the need for adjunctive treatments aimed at ameliorating dysbiosis, repairing the gut barrier, and suppressing inflammation. Table 2 summarizes the reported effects of microbiota-based adjunctive interventions in DCM animal models.

**Table 2:** The effects of microbiota-based adjunctive interventions in DCM animal models.

Animal model	Interventions	Duration of treatment	Core Conclusion
db/db mice	Probiotics was consists of ten Lactobacillus strains, orally fed with the probiotics once a day	6 weeks	Probiotics improved blood glucose and blood lipid parameters, increasing the levels of SCFAs-producing bacteria and SCFAs (98).
HFD/STZ-DCM	FMT, normal Ctrl mice were used as donors for FMT	2 weeks	FMT improves cardiac functions, decreases atrial natriuretic peptide (ANP) and B-type natriuretic peptide (BNP), alleviates cardiomyocyte hypertrophy and fibrosis of DCM mice (111).
C57BL			
HFD-C57Bl/6N mice	FMT was performed via oral gavage on three separate days (days 1, 3, 5)	6 weeks	FMT from nitrate-fed mice could prevent HFD–induced cardiac abnormalities, glucose intolerance, adipose inflammation, serum lipids, and gut dysbiosis (109).
HFD/STZ-DCM	Oral gavage of FMT, DCM mice pretreated with antibiotic cocktails and paeoniflorin were used as recipients	4 weeks	FMT improved cardiac function, alleviated myocardial fibrosis ratio, cardiomyocyte hypertrophy, decreased proportion of ferroptosis positive cells and myocardial injury markers (110).
C57BL			
HFD/STZ-DCM	FMT every two days, gut contents derived from control mice or myricetin and STZ treated mice	16 weeks	FMT had no differences in blood glucose and body weight, but alleviated cardiac dysfunction and fibrosis in DCM mice (47).
C57BL			
HFD/STZ-DCM	Mice were given BCAAs in their drinking water (10 mM leucine, 5 mM isoleucine and 5 mM valine)	12 weeks	Oral BCAAs supplementation induced mitochondrial damage and myocardial cell apoptosis and caused cardiac fibrosis and dysfunction in DCM mice (111).
C57BL			
HFD-C57BL mice	supplemented the SCFAs-containing diets with 5% SCFAs	30 weeks	SCFAs reduced body-fat gain, lowered hepatic triglycerides and improved insulin sensitivity (118).
HFD/STZ-DCM	deoxycholic acid (50 mg per kg body weight), taurocholic acid (200 mg per kg body weight) or vehicle (carboxymethyl cellulose) once daily	12 weeks	Administration of deoxycholic acid and taurocholic acid effectively alleviated lipid accumulation cardiac inflammation and oxidative stress, and cardiac remodelling in DCM mice (62).
C57BL			

HFD, high-fat diet; STZ, streptozotocin; FMT, fecal microbiota transplant; SCFAs, short-chain fatty acids; BCAAs, branched-chain amino acids.

### 4.1 Probiotics and prebiotics

Probiotics and prebiotics have been extensively investigated for enhancing the intestinal microecological balance by supplementing beneficial bacteria or promoting their growth, thereby potentially influencing DCM progression (96). Resistant starch, recognized as a prebiotic, has demonstrated significant potential in alleviating various diseases, altering the intestinal microbiota, and improving intestinal mucosal permeability towards a favorable direction in DCM (92). Probiotics can inhibit the proliferation of pathogenic flora such as Escherichia coli, Staphylococcus aureus, and Klebsiella, alter the composition of the gut microbiota, maintain a healthy intestinal microecological balance, and reduce inflammation levels (97, 98). Furthermore, probiotics can supply essential nutrients to beneficial bacteria, such as Bifidobacteria and Lactobacillus, stimulate their growth and activity, modulate the intestinal immune system, effectively enhance the local immune response, and mitigate intestinal inflammation (99, 100). Through fermentation, probiotics produce short-chain fatty acids that help maintain the acidic environment of the intestinal tract, which can inhibit the growth of pathogenic bacteria and promote the growth of the intestinal epithelium, thereby further augmenting their beneficial effects on the host (98).

Probiotic supplementation and dietary modulation constitute two complementary yet mechanistically divergent approaches to modulating the gut microbiota. Administration of probiotics, whether as single strains or defined microbial consortia, allows for precise dosing and may exert relatively rapid metabolic effects. Nevertheless, their therapeutic efficacy is highly strain-dependent, colonization is often transient, and outcomes are influenced by host-specific and ecological factors (101). By contrast, dietary modulation, commonly achieved through fermentable fiber or prebiotic intake, alters the luminal environment to promote the growth of resident SCFA-producing taxa (102). Although this strategy typically acts more gradually and requires sustained adherence, it has the potential to induce more persistent ecological remodeling. Clinically, a combined approach may be advantageous: probiotics can deliver short-term metabolic benefits, while concurrent fiber-based interventions reinforce microbial shifts and support long-term stability (103).

Ensuring safety remains a central challenge in microbiota-targeted therapies. While probiotics are generally considered safe in immunocompetent individuals, sporadic reports of bacteremia, fungemia, and even endocarditis have emerged, particularly among immunocompromised or critically ill patients (104). To minimize these risks, recommended strategies include employing genomically defined strains with resistance profiling, excluding or closely monitoring vulnerable populations, and maintaining vigilant surveillance for potential invasive infection (105). In contrast, dietary modulation is associated with a substantially lower risk of infection, though abrupt introduction may lead to gastrointestinal discomfort or metabolic disturbances (106). Collectively, these considerations highlight the importance of tailoring microbiota-directed interventions to both efficacy goals and patient safety profiles.

### 4.2 Fecal microbiota transplantation (FMT)

FMT involves transferring stool from a healthy donor to the recipient's gastrointestinal tract to restore the microbial balance (107). Although FMT is an innovative therapy for metabolic syndrome and cardiovascular disease (108), its use in DCM remains limited. Recently, Petrick et al. reported that DCM mice induced by an 8-week high-fat diet (HFD) developed left ventricular fibrosis, reduced stroke volume, glucose intolerance, adipose inflammation, and gut dysbiosis. Microbiota transplantation from nitrate-supplemented HFD mice or low-fat diet donors (*via* FMT administered on days 1, 3, and 5 post-modelling) reduced serum lipids and inflammation and prevented glucose intolerance and cardiac structural changes (109). Another study showed that FMT (administered every other day for 4 weeks) improved cardiac function, and suppressed ferroptosis in DCM mice (110). Zheng et al. also identified that FMT (2 weeks) improves cardiac functions, alleviates cardiomyocyte hypertrophy and fibrosis of DCM mice (111). Similarly, after treatment with antibiotics for 2 weeks, Zhu et al. found that FMT (every 2 days for 16 weeks) alleviated cardiac dysfunction and fibrosis, the inflammatory response of cardiomyocytes, and intestinal barrier integrity in streptozotocin-induced DCM mice (47). The above evidence has preliminarily confirmed the protective effect of FMT on DCM from preclinical studies.

In addition, Hu et al. found in clinical research that multiple FMT from lean donors in patients with obesity and related metabolic disorders could significantly reduce body weight, improve blood glucose and lipid levels, and achieve multiple metabolic benefits by reshaping the gut microbiota, reducing inflammation and TMAO levels, with good short-term safety (112). However, at present, this treatment is limited by the unstable colonization of probiotics and cannot be widely used, there are significant differences in baseline microbiota, genetic background and medication history among patients with different DCM, resulting in unstable therapeutic effects (113). Specifically, FMT effects depend heavily on donor microbiome, recipient baseline ecology, diet, and medications (especially antibiotics, proton pump inhibitors, statins). Many trials report transient engraftment and short-lived metabolic benefits. Predictable, durable engraftment in older, multimorbid DCM patients is uncertain (114). In addition, DCM is heterogeneous and microbiome modulation may help patients whose DCM has a metabolic/inflammatory component, but will likely be ineffective in purely genetic sarcomeric DCM. Trials must prespecify subgroups (e.g., the degree of intestinal flora imbalance, specific bacterial components and gut permeability markers) (115). More important, despite rigorous donor screening, there is a non-zero risk of transfer of pathogens or antimicrobial-resistant organisms. For immunocompromised or frail HF patients this risk is meaningful (116). In summary, while FMT theoretically holds promise for DCM treatment, its extended disease modeling period requires further investigation to determine the optimal intervention timing, frequency, and dosage.

### 4.3 Treatment with gut-derived metabolites

Gut-derived metabolites influence DCM by modulating metabolites produced by the gut microbiota. For instance, SCFAs such as acetic acid, propionic acid, and butyric acid have been shown to confer cardiovascular benefits by enhancing insulin sensitivity and mitigating inflammation (117, 118). Additionally, omega-3 fatty acids found in fish oil may enhance cardiovascular health and reduce myocardial inflammation (119). Compounds such as catechins in green tea and resveratrol in grapes have also been associated with improved cardiovascular function (120, 121). Adequate dietary fiber intake is crucial as it moderates postprandial blood glucose spikes and enhances insulin sensitivity, thanks to dietary fiber, short-chain fatty acids such as butyric acid are significantly increased (122), thereby reducing postprandial insulin requirements and contributing to more stable blood glucose levels (123). Stabilized blood glucose levels are instrumental in minimizing myocardial damage and safeguarding cardiac health, thereby decelerating DCM (124). Notably, Bile acids have been shown to mitigate cardiac inflammation and oxidative stress and enhance cardiac function by activating TGR5 receptors, thereby alleviating myocardial damage induced by diabetes (62). Future pharmacological research could concentrate on augmenting the synthesis of these advantageous metabolites or diminishing the production of detrimental metabolites as therapeutic strategies for DCM.

## 5 Conclusion

This review examines the intricate relationship between the gut microbiota and its metabolic processes in the context of DCM and proposes potential therapeutic strategies. Dysbiosis, intestinal metabolites such as bile acids and short-chain fatty acids, compromised intestinal barrier function, and inflammation induced by the gut microbiota have all been implicated in the pathogenesis of DCM. However, the precise mechanism underlying this association requires further investigation. This review underscores the potential for drug development focusing on probiotic- and metabolite-based interventions, as well as the regulation of systemic inflammation. These strategies may mitigate the progression of DCM by restoring intestinal microecological balance, enhancing intestinal barrier integrity, and attenuating inflammatory responses. Future research should aim to rigorously evaluate the efficacy and safety of these interventions in clinical settings with the goal of providing more effective cardiovascular protection for patients with DCM.
